# Assessment of DCE Utility for PCa Diagnosis Using PI-RADS v2.1: Effects on Diagnostic Accuracy and Reproducibility

**DOI:** 10.3390/diagnostics10030164

**Published:** 2020-03-17

**Authors:** Valentina Brancato, Giuseppe Di Costanzo, Luca Basso, Liberatore Tramontano, Marta Puglia, Alfonso Ragozzino, Carlo Cavaliere

**Affiliations:** 1 IRCCS SDN, 80143 Napoli, Italy; lbasso@sdn-napoli.it (L.B.); ltramontano@sdn-napoli.it (L.T.); ccavaliere@sdn-napoli.it (C.C.); 2 Ospedale S. Maria delle Grazie, 80078 Pozzuoli, Italy; giupe7700@yahoo.it (G.D.C.); martapuglia@alice.it (M.P.); alfonsoragozzino@gmail.com (A.R.)

**Keywords:** magnetic resonance imaging, prostate cancer, mpMRI, PI-RADS, DCE, reproducibility, ROC analysis

## Abstract

The role of dynamic contrast-enhanced-MRI (DCE-MRI) for Prostate Imaging-Reporting and Data System (PI-RADS) scoring is a controversial topic. In this retrospective study, we aimed to measure the added value of DCE-MRI in combination with T2-weighted (T2W) and diffusion-weighted imaging (DWI) using PI-RADS v2.1, in terms of reproducibility and diagnostic accuracy, for detection of prostate cancer (PCa) and clinically significant PCa (CS-PCa, for Gleason Score ≥ 7). 117 lesions in 111 patients were identified as suspicion by multiparametric MRI (mpMRI) and addressed for biopsy. Three experienced readers independently assessed PI-RADS score, first using biparametric MRI (bpMRI, including DWI and T2W), and then multiparametric MRI (also including DCE). The inter-rater and inter-method agreement (bpMRI- vs. mpMRI-based scores) were assessed by Cohen’s kappa (κ). Receiver operating characteristics (ROC) analysis was performed to evaluate the diagnostic accuracy for PCa and CS-PCa detection among the two scores. Inter-rater agreement was excellent for the three pairs of readers (κ ≥ 0.83), while the inter-method agreement was good (κ ≥ 0.73). Areas under the ROC curve (AUC) showed similar high-values (0.8 ≤ AUC ≤ 0.85). The reproducibility of PI-RADS v2.1 scoring was comparable and high among readers, without relevant differences, depending on the MRI protocol used. The inclusion of DCE did not influence the diagnostic accuracy.

## 1. Introduction

Multiparametric MRI (mpMRI) is an approach that involves the use of functional MRI methods, such as Diffusion Weighted Imaging (DWI) and Dynamic Contrast Enhanced (DCE) imaging, to supplement standard anatomical information provided by T1- and T2-weighted imaging [1,2].

The Prostate Imaging-Reporting and Data System (PI-RADS v1) score was developed and published in 2012 by the European Society of Urogenital Radiology (ESUR) in order to standardize the use of mpMRI in imaging PCa due to mpMRI expanding role in prostate cancer (PCa) diagnosis [2,3,4,5], mainly in patients with prior negative biopsies and/or increased PSA levels [6]. In its first version, for each MRI sequence a five-point scale was defined, which was based on the probability that the mpMRI findings were linked to the presence of a clinically significant PCa (CS-PCa) [6]. MRI sequences that were used to assess PI-RADS score were originally T2-weighted MRI (T2W), diffusion-weighted MRI (DWI), dynamic contrast-enhanced MRI (DCE-MRI), and proton MR spectroscopy (MRS). On the basis of PI-RADS v1 limitations, an updated PI-RADS version (PI-RADS v2) was developed in 2015 mainly due to the poor integration of the overall PI-RADS scoring leading to substantial variability in the system interpretation and use [7]. This update substantially strengthens the role of T2W and DWI MRI sequences, downgrading DCE-MRI contributes to a qualitative assessment through the presence/absence of focal enhancement [8].

When considering the large number of studies comparing the performances of PI-RADS v1 to PI-RADS v2 [8,9,10,11,12], there has been an increasing interest toward the use of contrast agents and bpMRI protocol for assessment of prostate lesions. Indeed, further studies have investigated the role of contrast injection in the PCa detection, when comparing biparametric MRI protocols (bpMRI) without DCE to mpMRI [13]. Moreover, several studies investigated PI-RADS v2 performances that were related to inter-reader variability and diagnostic accuracy in the detection of PCa and CS-PCa, also related to a less invasive bpMRI protocol [14,15,16,17,18]. Although the demonstrated improvements compared to the first version, especially in terms of standardization, diagnostic accuracy, and reproducibility [9,10], several inconsistencies, limitations, and conflicting results have been reported [14,15,16,17,18,19,20,21].

Therefore, a new update (PI-RADS v2.1) [22] has been recently released to overcome the above-mentioned drawbacks, improve inter-reader variability, and simplify the scoring assignment.

Modifications that are introduced in PI-RADS v2.1 should improve inter-reader variability and simplify PI-RADS scoring assessment, although, also in this version, the role of DCE-MRI/bpMRI is still regarded as very controversial and debated [22,23].

Because of the recent introduction of PI-RADS v2.1, studies evaluating its accuracy are few [24,25], and none of these aims to investigate the actual value of DCE for the PI-RADS assignment of PCa lesions.

The purpose of this study is to evaluate the added value of DCE MRI in combination with T2-weighted imaging and DWI while using PI-RADS v2.1 in terms of inter-reader reproducibility, bpMRI vs mpMRI comparison, and diagnostic accuracy for detection of PCa and CS-PCa using biopsy findings as the reference standard.

## 2. Materials and Methods

### 2.1. Patient Population

Patient imaging and histopathology records were collected at H.S. Maria delle Grazie, Italy. Informed consent was obtained before MR examination. 111 patients who underwent mpMRI of the prostate between April 2013 and September 2018 due to elevated PSA level and/or clinical suspicion of PCa and, subsequently, biopsy were included in this retrospective study.

### 2.2. Biopsy Protocol

All of the prostatic biopsies were TRUS-guided and performed while using an 18-gauge tru-cut needle, under anesthesia. Each patient underwent both systematic biopsy, with an average of 12 random samples of the entire prostate gland, and target biopsy, with at least three samples being taken from each lesion identified by MRI. The number of randomly taken samples could vary, depending on the dimensions of prostate gland, as well as the number of target samples could do, depending on the dimension of each lesion. Target sampling was performed with an MRI/TRUS fusion, alternately using the cognitive technique or dedicated software, coupled with ultrasound platforms from various companies.

### 2.3. Imaging Protocol

Routine clinical mpMRI acquisition includes T2W, DCE, and DWI. The DWI includes an apparent diffusion coefficient (ADC) map that was generated at the time of acquisition. Patients were injected with contrast agent Gadoteridol (Gd-HP-DO3A; ProHance, Bracco Diagnostics, Princeton, NJ, USA) with a dose of 0.1 mL/kg before MRI-DCE acquisition. All of the patients were imaged using MAGNETOM-Avanto scanner (Siemens Healthcare, Erlangen, Germany) scanner at 1.5 T with both endorectal coil and phase-array pelvic coil. Table 1 shows more details on the technical parameters of the MRI sequences.

### 2.4. MR Image Analysis and Interpretation Using PI-RADS v2.1

An evaluation of MR images was performed in two reading-sessions: the first reading-session was performed while considering a bpMRI protocol consisting of axial, sagittal, and coronal T2W images and axial DWI images with their corresponding ADC maps and a b-computed image with b = 1400 s/mm^2^; the second reading-session was performed after two weeks while considering the entire mpMRI protocol (T2W, DWI, and DCE-MRI), and rearranging patient IDs in a different order, to reduce memory bias. The mpMRI based image set and the bpMRI based image set were independently evaluated by three radiologists (R1, R2, and R3), respectively, with 10, seven, and eight years of experience, who were blinded to biopsy findings, according to the PI-RADS v2.1 scoring procedure described in the recently published guidelines. R2 was from a different institution than that where the mpMRI images were acquired and biopsies performed. Please note that, from now on, if PI-RADS version is not specified, we will refer to PI-RADS v2.1.

### 2.5. Statistical Analysis

All of the statistical analyses were performed using MedCalc software for Windows, version 19.0.3 (MedCalc Software, Ostend, Belgium) and, since several patients had more than one suspicion lesion, a per-lesion based approach was used, so each lesion was treated as separate case with its respective PI-RADS score. Analysis was first performed, regardless of the prostate zone in which the suspicion lesion was allocated. Subsequently, we focused on lesions that were located in peripheral zone (PZ), since only PIRADS 3 lesions in the PZ area should change the score, including DCE in MRI protocol. The inter-observer agreement was assessed for all of the pair-wise combinations of radiologists, firstly for the bpMRI-based PI-RADS scoring and then for the mpMRI-based one. Furthermore, the agreement between bpMRI-based and mpMRI-based PI-RADS scoring (inter-method agreement) was assessed for each radiologist. AN assessment of both kind of agreement was first performed while considering all lesions, and then only considering biopsy proven malignant lesions (GS ≥ 6), only GS ≥ 6 lesions located in PZ, only CS-PCa lesions (GS ≥ 7), and finally only considering nonmalignant lesions. Since PI-RADS score is an ordinal variable, the weighted Cohen’s kappa (κ) with linear weights was used to evaluate both inter-reader and inter-method agreement. The strength of agreement was evaluated as excellent if κ = 0.81–1.00, good if κ = 0.61–0.80, moderate if κ = 0.41–0.60, fair if κ = 0.21–0.40, and poor if κ = 0–0.20 [26]. A 95% CI for κ was also reported. The agreement rate was also computed, in order to evaluate the percentage of agreement between assignments.

The diagnostic performance of PI-RADS v2.1 in prostate cancer detection was assessed for bpMRI-based and mpMRI-based PI-RADS scoring assigned by each radiologist, while using the biopsy results as a reference standard and specifically considering as positive all lesions with a GS ≥ 6. The same diagnostic performance analysis was then performed while only considering GS ≥ 6 lesions located in PZ as positive, and then only CS-PCa lesions. The optimal threshold value (cutoff point) was found maximizing the Youden index and the value of area under the Receiver Operating Characteristic (ROC) curve (AUC) was analyzed to assess the capability of each type of PI-RADS v2.1 scoring (mpMRI-based, bpMRI-based), for each radiologist, of detecting PCa and CS-PCa. The values of *p* < 0.05 for AUC analysis mean that AUC is significantly different from 0.5, and so the assigned scoring has the ability to distinguish between PCa/nonPCa and CS-PCa/nonCS-PCa.

Analyzing the AUC using Z-test, and comparing the cutoff values and their related sensitivity and specificity was utilized to perform comparisons between diagnostic performances of mpMRI- and bpMRI-based PI-RADS (radiologist-based). These analyses were also carried out to compare the diagnostic performance of mpMRI and those of bpMRI, among the three radiologists. The values of *p* < 0.05 were considered to be statistically significant for Z-test.

## 3. Results

### 3.1. Patient Characteristics and Clinical Findings

Table 2 summarizes patients’ characteristics and biopsy results. The identified suspicion prostate lesions were 117, of which 78 classified as positive to PCa (GS ≥ 6). Among these 78 lesions, 41 were clinically significant (GS ≥ 7). Lesions that were negative to PCa, as well as all CS-PCa lesions, were all located in peripheral zone (PZ). Sixty-six of 78 PCa lesions were located in PZ.

### 3.2. Inter-Reader and Inter-Method Agreement

The different results, for each radiologist, of PI-RADS assignments and the related percentage of prostate lesions and clinically significant prostate lesions, are shown in Supplementary Table S1.

The inter-reader agreement between mpMRI-based PI-RADS was excellent for all three pairs of radiologists while considering all lesions. Similar results were obtained when considering all of the lesions positive to PCa and all PZ lesions positive to PCa, while κ increases when only considering CS-PCa lesions, in particular for the couple of R1–R2. When only considering lesions negative to biopsy, κ decreases for two pairs of radiologists, but remains high for the remaining couple. The inter-reader agreement between bpMRI-based PI-RADS scores remains excellent for all three pairs of radiologists when considering both all lesions and only lesion positive to PCa. Similar results were obtained when only CS-PCa lesions were considered, except for couple of R1–R3, for which κ slightly decreases. When only considering lesions that are negative to biopsy, κ decreases. Refer to Table 3 for inter-reader statistics and see Supplementary Materials (Tables S2–S25) for related crosstabs.

When considering all of the lesions, the assessment of agreement between mpMRI- and bpMRI-based PI-RADS revealed, for R1, R2, and R3, a κ value of 0.8, 0.86, and 0.85, respectively. When only considering PCa lesions (either as whole or considering only PZ PCa lesions) and then CS-PCa lesions, κ slightly decreases for R1 and remains quite similar for both R2 and R3, while agreement was excellent for R2 and R3 and good for R1 for lesions negative to biopsy. Refer to Table 4 for inter-method statistics and see Supplementary Materials (Tables S26–S40) for related crosstabs.

### 3.3. Diagnostic Accuracy

Despite different readers, different MRI protocols (mpMRI, bpMRI), and different positive classes (PCa, CS-PCa), the AUC values were similar.

The result of pairwise comparison of the mpMRI- and bpMRI-based PI-RADS scoring ROC curves was not significant, both considering PCa lesions and CS-PCa lesions as positive class (*p* > 0.05), meaning that there is no significant difference between the AUCs. Based on the Youden index, the best cutoff value results > 3 for all ROC analyses, except for mpMRI-based PI-RADS scoring by reader 3 for CS-PCa detection (Youden selected threshold > 4) and for bpMRI-based PI-RADS scoring by reader 2 for PCa detection (Youden selected threshold > 2). More noticeable differences were detected while examining sensitivity and specificity at threshold. Specifically, when considering mpMRI-based PI-RADS scoring, sensitivity and specificity were similar among all three readers for the detection of PCa and PZ PCa lesions, while, for CS-PCa detection, the sensitivity results increased for two readers and strongly decreased for the remaining one s compared to PCa detection analysis. Conversely, sensitivity results decreased for two readers and increased for the remaining one.

When considering bpMRI-based PI-RADS scoring, the sensitivity and specificity values substantially differ among all three readers for the detection of PCa, and these results showed a noticeable sensitivity decreasing and specificity increasing for R1 and R3 as compared to mpMRI-based PI-RADS findings, while, conversely, for R2 sensitivity value slightly increases and specificity decreases. The same behavior was observed for PZ PCa lesions.

**Figure 1:**
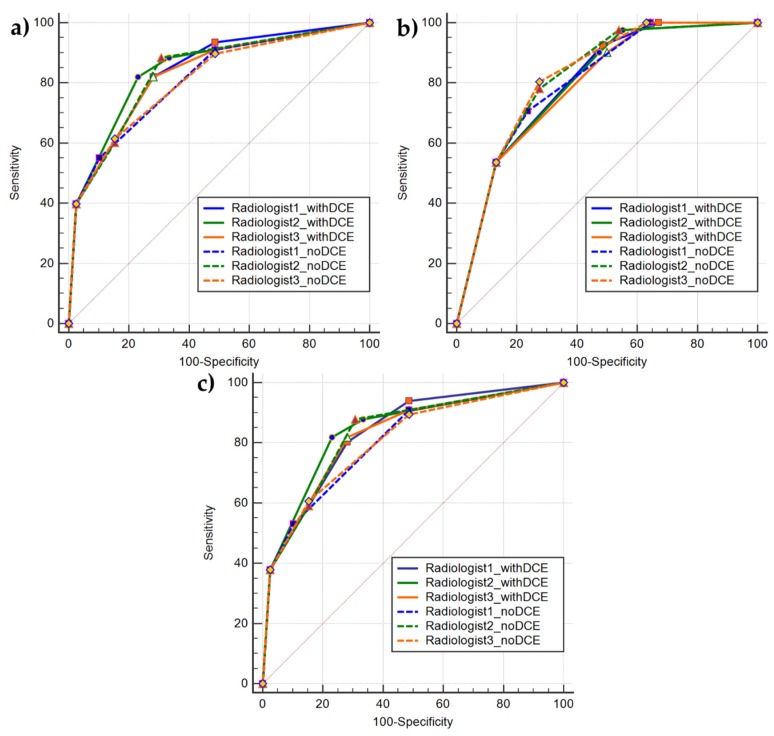
Comparison of ROC curves showing diagnostic accuracy of mpMRI-based (continuous lines) and bpMRI-based (dashed line) PI-RADS v2.1 scoring for the three readers: (**a**) ROC curves showing diagnostic accuracy for PCa detection; (**b**) ROC curves showing diagnostic accuracy for CS-PCa detection. Points, triangles, squares and diamonds correspond to PI-RADS thresholds; and, (**c**) ROC curves showing diagnostic accuracy for PCa detection in peripheral zone (PZ). Please note that for diagnostic accuracy analysis in PZ PCa lesion detection, the total sample consisted of 105 PZ lesions, of which 66 positive to biopsy.

For CS-PCa detection, the sensitivity values differ slightly among the three readers, while those of specificity were similar among all three readers. The sensitivity results increased for R1 and R3 and decreased for R2 when compared to PCa detection results. Conversely, the specificity results slightly increased for R2 and decreased for the remaining two. Moreover, these results showed a noticeable sensitivity decreasing and specificity increasing for R1 and R3 when compared to mpMRI-based PI-RADS findings, while, conversely, for R2 sensitivity value slightly increases and specificity decreases. Table 5 summarizes all of the results of ROC analyses. Please note that, for diagnostic accuracy analysis in PZ PCa lesion detection, the total sample consisted of 105 PZ lesions, of which 66 were positive to biopsy. Figure 1 shows ROC curves for PCa detection, PZ PCa detection, and CS-PCa detection.

## 4. Discussion

In this study, we evaluated PI-RADS v2.1 scoring in terms of the detection of PCa, primarily focusing on the role of DCE-MRI and on the efficacy of bpMRI assessment on inter-reader variability and diagnostic accuracy.

Using biopsy findings as reference standard, we first investigated the differences in inter-reader agreement among three radiologists that were blinded to each other assignments and biopsy results. Subsequently, we focused on inter-method agreement, in order to assess, with an intra-reader analysis, the agreement between mpMRI- and bpMRI-based PI-RADS scoring. Finally, we focused on diagnostic performance of PI-RADS v2.1 scoring when assigned on the basis of mpMRI protocol and when assigned on the basis of a bpMRI protocol for PCa and CS-PCa detection. We considered it appropriate to perform a sub-analysis on lesions in the peripheral zone (PZ), since only PIRADS 3 lesions in the PZ area should change the score, including DCE in MRI protocol.

Inter-reader agreement for both PCa, PZ PCa, and CS-PCa (all in PZ in our sample) detection was remarkably higher than that for lesions negative to biopsy. This might be due to the heterogeneous appearance of benign lesions, such as prostatitis and benign prostatic hyperplasia (BPH). The best overall results for inter-rater agreement were reached when only considering CS-PCa lesions and a mpMRI-based PI-RADS classification.

Concerning the detection of PCa, PZ PCa, and CS-PCa lesions, the κ values were comparable overall and extremely high, without relevant differences, depending on the inclusion of DCE in MRI protocol used.

Previous studies using PI-RADS v2 assessment found κ values that were lower than those found in our study [15,17,27]. However, since the κ values depend on many factors (including the prevalence of disease) [28], it makes no sense to compare κ value obtained in different studies. To account for this, PI-RADS v2 and 2.1 scoring should both be assigned on the same group of patients, as done in recent studies by Tamada et al. and Byun et al. [24,25].

Regarding inter-method agreement, despite estimated κ values and agreement rates were, respectively, higher than 0.85 and 70.5% (regardless of the reader and the examined kind of lesions), it should be noted that the main source of disagreement lies in the different number of lesions that were classified as PI-RADS 3. Focusing on PZ lesions scored as PIRADS 3 in bpMRI reading session, 72%, 79% and 84,2% of lesions scored as PIRADS 3 in bpMRI reading session, respectively, by radiologist 1, 2, and 3, were scored as PIRADS 4 in the mpMRI session (see Tables S38–S40 added in Supplementary Materials).

Accordingly, when considering the inter-method agreement analysis, the omission of DCE in mpMRI protocol led to an increasing number of PI-RADS 3 scored lesions. This effect was already reported for PI-RADS v2, both in heterogeneous samples [29] then in selected PCa lesions [16].

Regarding diagnostic accuracy, in ROC curve analysis, there was no significant difference regarding the AUC among readers or between different MRI protocols that were used to score lesions. Nevertheless, the ranges of sensitivity and specificity for PCa detection using mpMRI were more contained (sen = 0.82; spec = 0.72–0.77) with respect to bpMRI values (sen = 0.55–0.88; spec = 0.69–0.9). Conversely, for the detection of CS-PCa using mpMRI, sensitivity and specificity values have a wider range for mpMRI assessment (sen = 0.54–0.92; spec = 0.51–0.87), than bpMRI evaluation (sen = 0.71–0.8; spec = 0.72–0.76).

It should be noted that the adoption of PI-RADS v2.1 adjustments should not affect the overall diagnostic accuracy when compared to PI-RADS v2, but it should improve inter-reader variability and simplify score assignment [22]. Hence, it makes sense for us to compare these results with studies regarding diagnostic accuracy for PCa detection performed using PI-RADS v2. For the detection of PCa, regardless of GS, the observed sensitivity and specificity values fall within the ranges indicated in a diagnostic test accuracy meta-analysis by Kang et al. [30], including 10 studies with population characteristics that were similar to those considered in our study and using a per-lesion approach, and verifying that bpMRI and mpMRI had similar diagnostic performances for PCa detection.

The strengths of our study are that, unlike many other studies performing analyses using a patient-based approach, we performed a per-lesion based analysis; in addition to other studies assessing intra-reader differences between bpMRI- and mpMRI-based PI-RADS simply visually inspecting assigned scores, we also performed an inter-method analysis using κ.

Some limitations existed in our study. First of all, the data collection according to retrospective study design is prone to introduce bias, such as selection bias and information bias [31]. Subsequently, due to the small samples in case of TZ and CZ lesions (respectively, five and seven lesions), we could not perform sub-analyses in these zones. Even if, according to PIRADS v2.1, the DCE findings should have low impact on PI-RADS scoring for the TZ and CZ lesion, it could be interesting to assess if, when comparing bpMRI- and mpMRI-based, discrepancies in inter-reader agreement, diagnostic performances, and percentage of intra-reader disagreement among the two methods, could be detected. Moreover, the effects of reader experience on diagnostic performance, inter-observer, and inter-method agreement were not examined. Gatti et al. [17] have found that the omission of DCE from mpMRI protocol does not affect PI-RADS v2 diagnostic performance only when readers are experts, affecting ROC analysis statistics.

Conversely, Di Campli et al. [18] showed that the experience of the reader does not significantly affect the diagnostic performance of bpMRI and mpMRI protocols. In our study, although the three readers shared similar years of experience on prostate imaging, one of them (R2) belonged to another institution, with a different scanner, magnetic field, and acquisition protocol. Nevertheless, similar performances were recorded in PI-RADS scoring.

The use of biopsy instead of radical prostatectomy specimens as the reference standard can also be considered to be a limitation. The use of prostatectomy specimens could allow for more accurate anatomic correlation with MR images and the correct assessment of GS [14].

Finally, the lacking of studies investigating the value of DCE for PI-RADS v2.1 scoring assignment prevented us to perform more appropriate comparisons.

## 5. Conclusions

The reproducibility of PI-RADS v2.1 scoring for the detection of both PCa and CS-PCa was comparable and high among readers, without relevant differences, depending on the inclusion of DCE in MRI protocol.

Findings that were related to diagnostic accuracy revealed that PI-RADS v2.1 scoring assigned on bpMRI protocol results in being comparable with that assigned on the mpMRI protocol.

Finally, although DCE in mpMRI protocol determines longer examination times, more elevated costs, and possible collateral effects/contraindications to contrast agents [32], its value can be crucial in the case of very suggestive clinical history for PCa, prior negative biopsies, and unclear findings from bpMRI. For these reasons, although we did not detect a clear added value of DCE in terms of reproducibility and diagnostic accuracy, we recommend considering it as an additional sequence, depending on the kind of lesion and the clinical characteristics of the patient.

## Figures and Tables

**Table 1:** Parameters of mpMRI sequences. TR = Repetition Time; TE = Echo Time; ST = Slice Thickness; Avg. = Averages; BW = Bandwidth; FOV = Field of view; and, FA = Flip angle.

Sequence	Slices	TR (msec)	TE (msec)	ST (mm)	Avg.	BW	Matrix	FOV (mm)	FA
T2W Sagittal	9	4740	102	3	2	200	320 × 310	200 × 200	137
T2 Axial	23	5610	102	3	2	200	320 × 272	200 × 200	123
T2 Coronal	16	4000	102	3	1	200	320 × 310	211 × 211	138
T2 Lymph nodes	36	7620	98	6.5	1	130	512 × 247	341 × 390	135
DWI ^a^	19	3505	75	3	6	1698	128 × 128	250 × 250	90
T1 vibe tra FA	20	5.5	2.34	3.5	8	300	320 × 112	208 × 417	2
T1 vibe tra FA	20	5.5	2.34	3.5	8	300	320 × 112	208 × 417	15
T1 vibe dyn ^b^	22	5.5	2.34	3.5	1	300	320 × 112	208 × 417	10

^a^ DWI performed with b-values of 50, 400 and 1000 s/mm^2^. ^b^ with 32 measurements.

**Table 2:** Clinical details and biopsy results on a per-patient and per-lesion basis. B PSA = Prostate-Specific Antigen; PCa = prostate cancer; CS-PCa = clinically significant prostate cancer (defined as Gleason score ≥ 7).

Variable	Value (Patient Based)	Value (Lesion Based)
**Clinical Variables**		
No. of patients/lesions [*n*]	111	117
Median age [y (range)]	69 (50–81)	
Mean PSA density [ng/mL^2^]	0.26	
Prostate volume [cm^3^]	57.5	
Prostatic zone		
PZ		105 (89.7)
TZ		5 (4.3)
CZ		7 (6)
**Biopsy Results**		
PCa not detected [*n* (%)]	39 (35.1)	39 (33.3)
PCa detected [*n* (%)]	72 (64.9)	78 (66.7)
CS-PCa detected [*n* (%)]	38 (34.2)	41 (35)
Gleason scores for CS-PCa lesions [*n* (%)]		
3 + 4		6 (5.1)
4 + 3		15 (12.8)
4 + 4		15 (12.8)
4 + 5		3 (2.5)
5 + 3		1 (0.8)
5 + 4		1 (0.8)

**Table 3:** Inter-reader agreement results: agreement rate and κ-value for each couple of readers in assignment of Prostate Imaging-Reporting and Data System (PI-RADS) v2.1 score. Numbers in round brackets are ratios between the number of lesions assessed with the same PI-RADS score from both readers and the total number of lesions. Numbers in square brackets are 95% confidence interval lower and upper bounds for κ-value. DCE = dynamic contrast-enhanced MRI; PCa = prostate cancer; PZ = peripheral zone; CS-PCa = clinically significant prostate cancer (defined as Gleason score > 3 + 3); R1-R2 = reader 1 and reader 2; R1-R3 = reader 1 and reader 3; R2-R3 = reader 2 and reader 3.

PI-RADS v2.1 Type	Lesion Type	Agreement Rate, % [Number of Lesions in Agreement/Total No. of Lesions]			κ Value [95% CI]		
		R1-R2	R1-R3	R2-R3	R1-R2	R1-R3	R2-R3
with DCE	All lesions (*n* = 117)	85.5 (100/117)	91.4 (107/117)	90 (106/117)	0.87 [0.82–0.93]	0.92 [0.89–0.97]	0.92 [0.87–0.97]
no DCE	All lesions (*n* = 117)	82 (96/117)	86.3 (101/117)	92.3 (108/117)	0.86 [0.8–0.92]	0.89 [0.84–0.94]	0.94 [0.9–0.98]
with DCE	PCa lesions (*n* = 78)	87.1 (68/78)	92.3 (72/78)	94.9 (74/78)	0.86 [0.79–0.94]	0.92 [0.85–0.98]	0.95 [0.9–1.00]
no DCE	PCa lesions (*n* = 78)	87.2 (68/78)	87.2 (68/78)	97.4 (76/78)	0.89 [0.82–0.95]	0.88 [0.82–0.95]	0.98 [0.95–1.00]
with DCE	PZ PCa lesions (*n* = 66)	86.4 (57/66)	92.4 (61/66)	93.9 (62/66)	0.86 [0.77–0.94]	0.92 [0.85–0.99]	0.94 [0.89–1.00]
no DCE	PZ PCa lesions (*n* = 66)	87.9 (58/66)	87.9 (58/66)	97.4 (64/66)	0.9 [0.83–0.97]	0.89 [0.82–0.97]	0.97 [0.94–1.00]
with DCE	CS-PCa lesions (*n* = 41)	95.1 (39/41)	97.6 (40/41)	97.6 (40/41)	0.93 [0.84–1.00]	0.96 [0.89–1.00]	0.96 [0.9–1.00]
no DCE	CS-PCa lesions (*n* = 41)	85.4 (35/41)	85.4 (35/41)	92.1 (39/41)	0.84 [0.73–0.95]	0.83 [0.71–0.95]	0.94 [0.87–1.00]
with DCE	non-PCa lesions (*n* = 39)	82 (32/39)	89.7 (35/39)	82 (32/39)	0.78 [0.62–0.94]	0.89 [0.79–0.99]	0.78 [0.62–0.94]
no DCE	non-PCa lesions (*n* = 39)	71.8 (28/39)	84.6 (33/39)	82 (32/39)	0.63 [0.44–0.83]	0.8 [0.65–0.95]	0.78 [0.61–0.94]

**Table 4:** Inter-method agreement results: agreement rate between mpMRI- and bpMRI-based PI-RADS score for each radiologist. Numbers in round brackets are ratios between the number of lesions assessed from the reader with the same PI-RADS score using the two approaches and the total number of lesions. Numbers in square brackets are 95% confidence interval lower and upper bounds for κ-value. PCa = prostate cancer; PZ = peripheral zone; CS-PCa = clinically significant prostate cancer (defined as Gleason score > 3 + 3); R1 = reader 1; R2 = reader 2; R3 = reader 3.

Lesion Type	Agreement Rate, % [Number of Lesions in Agreement/Total No. of Lesions]			κ Value [95% CI]		
	R1	R2	R3	R1	R2	R3
All lesions (*n* = 117)	74.3 (87/117)	82 (96/117)	81.2 (95/117)	0.8 [0.73–0.87]	0.86 [0.8–0.92]	0.85 [0.79–0.91]
PCa lesions (*n* = 78)	70.5 (55/78)	78.2 (61/78)	78.2 (61/78)	0.73 [0.63–0.82]	0.8 [0.72–0.89]	0.8 [0.71–0.88]
PZ PCa lesions (*n* = 66)	69.7 (46/66)	77.3 (51/66)	77.3 (51/66)	0.72 [0.63–0.82]	0.8 [0.7–0.89]	0.79 [0.69–0.88]
CS-PCa lesions (*n* = 41)	78 (32/41)	87.8 (36/41)	90.2 (37/41)	0.73 [0.6–0.86]	0.85 [0.73–0.97]	0.87 [0.76–0.98]
non-PCa lesions (*n* = 39)	82 (32/39)	89.7 (35/39)	87.2 (34/39)	0.79 [0.67–0.92]	0.87 [0.75–0.98]	0.86 [0.74–0.97]

**Table 5:** Diagnostic accuracy of mpMRI- and bpMRI-based PI-RADS in detection of PCa and CS-PCa for each radiologist: Area under the Receiver Operating Characteristic (ROC) curve (AUC), selected threshold according to the maximum Youden’s index, sensitivity and specificity at threshold. Numbers in square brackets are 95% confidence interval lower and upper bounds for AUC. DCE = dynamic contrast-enhanced MRI; Pos class = positive class; PCa = prostate cancer; PZ = peripheral zone; CS-PCa = clinically significant prostate cancer (defined as Gleason score > 3 + 3); th = threshold; R1 = reader 1; R2 = reader 2; R3 = reader 3. Please note that for diagnostic accuracy analysis in PZ PCa lesion detection, the total sample consisted of 105 PZ lesions, of which 66 positive to biopsy.

PI-RADS v2.1 Type	Pos Class	AUC [95% CI]			Youden Selected th			Sen at th			Specificity at th		
		R1	R2	R3	R1	R2	R3	R1	R2	R3	R1	R2	R3
with DCE	PCa	0.84 [0.86–0.9]	0.85 [0.77–0.91]	0.82 [0.75–0.89]	>3	>3	>3	0.80	0.82	0.82	0.72	0.77	0.72
with DCE	PCa (PZ)	0.83 [0.75–0.9]	0.84 [0.76–0.91]	0.82 [0.74–0.89]	>3	>3	>3	0.82	0.82	0.82	0.72	0.77	0.72
with DCE	CS-PCa	0.8 [0.72–0.87]	0.8 [0.71–0.87]	0.8 [0.71–0.86]	>3	>3	>4	0.92	0.9	0.54	0.51	0.53	0.87
no DCE	PCa	0.81 [0.73–0.88]	0.84 [0.76–0.9]	0.8 [0.73–0.88]	>3	>2	>3	0.55	0.88	0.61	0.9	0.69	0.85
no DCE	PCa (PZ)	0.81 [0.72–0.88]	0.83 [0.75–0.9]	0.8 [0.72–0.88]	>3	>2	>3	0.53	0.88	0.61	0.9	0.69	0.85
no DCE	CS-PCa	0.8 [0.72–0.87]	0.82 [0.73–0.88]	0.82 [0.74–0.89]	>3	>3	>3	0.71	0.78	0.8	0.76	0.72	0.72

## Supplementary Materials

The following are available online at https://www.mdpi.com/2075-4418/10/3/164/s1.
