# Supplementary material for: Assessment of DCE Utility for PCa Diagnosis Using PI-RADS v2.1: Effects on Diagnostic Accuracy and Reproducibility
Source: Diagnostics (Basel). 2020 Mar 17;10(3):164. doi: 10.3390/diagnostics10030164 (PMC7151226; doi:10.3390/diagnostics10030164)
Supplement: Supplementary file 1 [file diagnostics-10-00164-s001.pdf]

Supplementary Materials

Scores of mpMRI-based PI-RADS and bpMRI-based PI-RADS

The three readers classified prostate lesions according to PI-RADS scoring in different proportions, except for PI-RADS scores of 1 and 5, which were assigned respectively to 0 and 32 lesions by all 3 radiologists, using either mpMRI protocol or bpMRI protocol. Using mpMRI, PI-RADS scores of 2, 3, and 4 were respectively attributed to 25, 17 and 43 lesions by reader 1; 35, 9, 41 lesions by reader 2; 27, 15 and 43 lesions by reader 3. Omitting DCE, PI-RADS scores of 2, 3, and 4 were respectively attributed to 27, 43 and 15 lesions by reader 1; 36, 28, 21 lesions by reader 2; 28, 35 and 22 lesions by reader 3. Immediately evident is the higher variability of PI-RADS scored as 3 among the radiologists. Moreover, a marked increase of lesions attributed to a PI-RADS score of 3, at the expense to a decrease of lesions attributed to a PI-RADS score of 4, was evident without the inclusion of DCE in the PI-RADS scoring.

**PI-RADS v2.1 score**	**mpMRI-based**									**bpMRI-based**								
	**R1**			**R2**			**R3**			**R1**			**R2**			**R3**		
	**All (%)**	**n PCa (%)**	**n CS-PCa (%)**	**All (%)**	**n PCa (%)**	**n CS-PCa (%)**	**All (%)**	**n PCa (%)**	**n CS-PCa (%)**	**All (%)**	**n PCa (%)**	**n CS-PCa (%)**	**All (%)**	**n PCa (%)**	**n CS-PCa (%)**	**All (%)**	**n PCa (%)**	**n CS-PCa (%)**
**1**	0 (0)	0 (0)	0 (0)	0 (0)	0 (0)	0 (0)	0 (0)	0 (0)	0 (0)	0 (0)	0 (0)	0 (0)	0 (0)	0 (0)	0 (0)	0 (0)	0 (0)	0 (0)
**2**	25 (21.3)	5 (4.3)	0 (0)	35 (29.9)	9 (7.7)	1 (0.8)	27 (23.1)	7 (6)	0 (0)	27 (23.1)	7 (6)	0 (0)	36 (30.8)	9 (7.7)	1 (0.8)	28 (23.9)	8 (6.8)	0 (0)
**3**	17 (14.5)	9 (7.7)	3 (2.6)	9 (7.7)	5 (4.3)	3 (2.6)	15 (12.8)	7 (6)	4 (3.4)	43 (36.8)	28 (23.9)	12 (10.3)	28 (23.9)	22 (18.8)	8 (6.8)	35 (29.9)	22 (18.8)	8 (6.8)
**4**	43 (36.8)	33 (28.2)	16 (13.7)	41 (35)	33 (28.2)	15 (12.8)	43 (36.8)	33 (28.2)	15 (12.8)	15 (12.8)	12 (10.3)	7 (6)	21 (17.9)	16 (13.7)	10 (8.5)	22 (18.8)	17 (14.5)	11 (9.4)
**5**	32 (27.4)	31 (26.5)	22 (18.8)	32 (27.4)	31 (26.5)	22 (18.8)	32 (27.4)	31 (26.5)	22 (18.8)	32 (27.4)	31 (26.5)	22 (18.8)	32 (27.4)	31 (26.5)	22 (18.8)	32 (27.4)	31 (26.5)	22 (18.8)
**Total**	117 (100)	78 (66,7)	41 (35)	117 (100)	78 (66,7)	41 (35)	117 (100)	78 (66,7)	41 (35)	117 (100)	78 (66,7)	41 (35)	117 (100)	78 (66,7)	41 (35)	117 (100)	78 (66,7)	41 (35)

Table S1: PI-RADS v2.1 scores assigned to each of the 117 lesions by each radiologist using both an mpMRI-based approach and a bpMRI-based one, with relative rates (number and percentage) of PCa lesions and CS-PCa lesions. "All" stands for All PCa lesions; PCa = prostate cancer; CS-PCa = clinically significant prostate cancer (defined as Gleason score > 3+3); R1 = reader 1; R2 = reader 2; R3 = reader 3.

Crosstabs for inter-reader agreement

Crosstabs for mpMRI-based PI-RADS v2.1 considering all lesions (n = 117)

**Radiologist2_withDCE**	**Radiologist1_withDCE**				**Total (%)**
	**2**	**3**	**4**	**5**	
**2**	25	9	1	0	35 (29,9%)
**3**	0	5	4	0	9 (7,7%)
**4**	0	3	38	0	41 (35,0%)
**5**	0	0	0	32	32 (27,4%)
**Total (%)**	25 (21,4%)	17 (14,5%)	43 (36,8%)	32 (27,4%)	117

Table S2: Crosstab for inter-reader agreement between radiologist 1 and radiologist 2.

**Radiologist3_withDCE**	**Radiologist1_withDCE**				**Total (%)**
	**2**	**3**	**4**	**5**	
**2**	24	3	0	0	27 (23,1%)
**3**	1	11	3	0	15 (12,8%)
**4**	0	3	40	0	43 (36,8%)
**5**	0	0	0	32	32 (27,4%)
**Total (%)**	25 (21,4%)	17 (14,5%)	43 (36,8%)	32 (27,4%)	117

Table S3: Crosstab for inter-reader agreement between radiologist 1 and radiologist 3.

**Radiologist3_withDCE**	**Radiologist2_withDCE**				**Total (%)**
	**2**	**3**	**4**	**5**	
**2**	27	0	0	0	27 (23,1%)
**3**	7	7	1	0	15 (12,8%)
**4**	1	2	40	0	43 (36,8%)
**5**	0	0	0	32	32 (27,4%)
**Total (%)**	35 (29,9%)	9 (7,7%)	41 (35,0%)	32 (27,4%)	117

Table S4: Crosstab for inter-reader agreement between radiologist 2 and radiologist 3.

Crosstabs for bpMRI-based PI-RADS v2.1 considering all lesions (n = 117)

	Radiologist1_noDCE				Total (%)
**Radiologist2_noDCE**	**2**	**3**	**4**	**5**	
**2**	27	9	0	0	36 (30,8%)
**3**	0	25	3	0	28 (23,9%)
**4**	0	9	12	0	21 (17,9%)
**5**	0	0	0	32	32 (27,4%)
**Total (%)**	27 (23,1%)	43 (36,8%)	15 (12,8%)	32 (27,4%)	117

Table S5: Crosstab for inter-reader agreement between radiologist 1 and radiologist 2.

	Radiologist1_noDCE				Total (%)
**Radiologist3_noDCE**	**2**	**3**	**4**	**5**	
**2**	26	2	0	0	28 (23,9%)
**3**	1	31	3	0	35 (29,9%)
**4**	0	10	12	0	22 (18,8%)
**5**	0	0	0	32	32 (27,4%)
**Total (%)**	27 (23,1%)	43 (36,8%)	15 (12,8%)	32 (27,4%)	117

Table S6: Crosstab for inter-reader agreement between radiologist 1 and radiologist 3.

	Radiologist2_noDCE				Total (%)
**Radiologist3_noDCE**	**2**	**3**	**4**	**5**	
**2**	28	0	0	0	28 (23,9%)
**3**	8	27	0	0	35 (29,9%)
**4**	0	1	21	0	22 (18,8%)
**5**	0	0	0	32	32 (27,4%)
**Total (%)**	36 (30,8%)	28 (23,9%)	21 (17,9%)	32 (27,4%)	117

Table S7: Crosstab for inter-reader agreement between radiologist 2 and radiologist 3.

Crosstabs for mpMRI-based PI-RADS v2.1 considering all PCa lesions (n = 78)

	Radiologist1_withDCE				Total (%)
**Radiologist2_withDCE**	**2**	**3**	**4**	**5**	
**2**	5	4	0	0	9 (11,5%)
**3**	0	2	3	0	5 (6,4%)
**4**	0	3	30	0	33 (42,3%)
**5**	0	0	0	31	31 (39,7%)
**Total (%)**	5 (6,4%)	9 (11,5%)	33 (42,3%)	31 (39,7%)	78

Table S8: Crosstab for inter-reader agreement between radiologist 1 and radiologist 2.

	Radiologist1_withDCE				Total (%)
**Radiologist3_withDCE**	**2**	**3**	**4**	**5**	
**2**	5	2	0	0	7 (9,0%)
**3**	0	5	2	0	7 (9,0%)
**4**	0	2	31	0	33 (42,3%)
**5**	0	0	0	31	31 (39,7%)
**Total (%)**	5 (6,4%)	9 (11,5%)	33 (42,3%)	31 (39,7%)	78

Table S9: Crosstab for inter-reader agreement between radiologist 1 and radiologist 3.

	Radiologist2_withDCE				Total (%)
**Radiologist3_withDCE**	**2**	**3**	**4**	**5**	
**2**	7	0	0	0	7 (9,0%)
**3**	2	4	1	0	7 (9,0%)
**4**	0	1	32	0	33 (42,3%)
**5**	0	0	0	31	31 (39,7%)
**Total (%)**	9 (11,5%)	5 (6,4%)	33 (42,3%)	31 (39,7%)	78

Table S10: Crosstab for inter-reader agreement between radiologist 2 and radiologist 3.

Crosstabs for bpMRI-based PI-RADS v2.1 considering all PCa lesions (n = 78)

	Radiologist1_noDCE				Total (%)
**Radiologist2_noDCE**	**2**	**3**	**4**	**5**	
**2**	7	2	0	0	9 (11,5%)
**3**	0	20	2	0	22 (28,2%)
**4**	0	6	10	0	16 (20,5%)
**5**	0	0	0	31	31 (39,7%)
**Total (%)**	7 (9,0%)	28 (35,9%)	12 (15,4%)	31 (39,7%)	78

Table S11: Crosstab for inter-reader agreement between radiologist 1 and radiologist 2.

	Radiologist1_noDCE				Total (%)
**Radiologist3_noDCE**	**2**	**3**	**4**	**5**	
**2**	7	1	0	0	8 (10,3%)
**3**	0	20	2	0	22 (28,2%)
**4**	0	7	10	0	17 (21,8%)
**5**	0	0	0	31	31 (39,7%)
**Total (%)**	7 (9,0%)	28 (35,9%)	12 (15,4%)	31 (39,7%)	78

Table S12: Crosstab for inter-reader agreement between radiologist 1 and radiologist 3.

	Radiologist2_noDCE				Total (%)
**Radiologist3_noDCE**	**2**	**3**	**4**	**5**	
**2**	8	0	0	0	8 (10,3%)
**3**	1	21	0	0	22 (28,2%)
**4**	0	1	16	0	17 (21,8%)
**5**	0	0	0	31	31 (39,7%)
**Total (%)**	9 (11,5%)	22 (28,2%)	16 (20,5%)	31 (39,7%)	78

Table S13: Crosstab for inter-reader agreement between radiologist 2 and radiologist 3.

Crosstabs for mpMRI-based PI-RADS v2.1 considering clinically significant PCa lesions (n = 41)

	Radiologist1_withDCE				Total (%)
Radiologist2_withDCE	2	3	4	5	
2	0	1	0	0	1 (2,4%)
3	0	2	1	0	3 (7,3%)
4	0	0	15	0	15 (36,6%)
5	0	0	0	22	22 (53,7%)
Total (%)	0 (0,0%)	3 (7,3%)	16 (39,0%)	22 (53,7%)	41

Table S14: Crosstab for inter-reader agreement between radiologist 1 and radiologist 2.

	Radiologist1_withDCE			Total (%)
Radiologist3_withDCE	3	4	5	
3	3	1	0	4 (9,8%)
4	0	15	0	15 (36,6%)
5	0	0	22	22 (53,7%)
Total (%)	3 (7,3%)	16 (39,0%)	22 (53,7%)	41

Table S15: Crosstab for inter-reader agreement between radiologist 1 and radiologist 3.

	Radiologist2_withDCE				Total (%)
Radiologist3_withDCE	2	3	4	5	
2	0	0	0	0	0 (0,0%)
3	1	3	0	0	4 (9,8%)
4	0	0	15	0	15 (36,6%)
5	0	0	0	22	22 (53,7%)
Total (%)	1 (2,4%)	3 (7,3%)	15 (36,6%)	22 (53,7%)	41

Table S16: Crosstab for inter-reader agreement between radiologist 2 and radiologist 3.

Crosstabs for bpMRI-based PI-RADS v2.1 considering clinically significant PCa lesions (n = 41)

	**Radiologist1_noDCE**				**Total (%)**
**Radiologist2_noDCE**	**2**	**3**	**4**	**5**	
2	0	1	0	0	1 (2,4%)
3	0	7	1	0	8 (19,5%)
4	0	4	6	0	10 (24,4%)
5	0	0	0	22	22 (53,7%)
**Total (%)**	0 (0,0%)	12 (29,3%)	7 (17,1%)	22 (53,7%)	41

Table S17: Crosstab for inter-reader agreement between radiologist 1 and radiologist 2.

	**Radiologist1_noDCE**			**Total (%)**
**Radiologist3_noDCE**	**3**	**4**	**5**	
**3**	7	1	0	8 (19,5%)
**4**	5	6	0	11 (26,8%)
**5**	0	0	22	22 (53,7%)
**Total (%)**	12 (29,3%)	7 (17,1%)	22 (53,7%)	41

Table S18: Crosstab for inter-reader agreement between radiologist 1 and radiologist 3.

	**Radiologist2_noDCE**				**Total (%)**
**Radiologist3_noDCE**	**2**	**3**	**4**	**5**	
**2**	0	0	0	0	0 (0,0%)
**3**	1	7	0	0	8 (19,5%)
**4**	0	1	10	0	11 (26,8%)
**5**	0	0	0	22	22 (53,7%)
**Total (%)**	1 (2,4%)	8 (19,5%)	10 (24,4%)	22 (53,7%)	41

Table S19: Crosstab for inter-reader agreement between radiologist 2 and radiologist 3.

Crosstabs for mpMRI-based PI-RADS v2.1 considering non-PCa lesions (n = 39)

	Radiologist1_withDCE				Total (%)
**Radiologist2_withDCE**	**2**	**3**	**4**	**5**	
**2**	20	5	1	0	26 (66,7%)
**3**	0	3	1	0	4 (10,3%)
**4**	0	0	8	0	8 (20,5%)
**5**	0	0	0	1	1 (2,6%)
**Total (%)**	20 (51,3%)	8 (20,5%)	10 (25,6%)	1 (2,6%)	39

Table S20: Crosstab for inter-reader agreement between radiologist 1 and radiologist 2.

	Radiologist1_withDCE				Total (%)
**Radiologist3_withDCE**	**2**	**3**	**4**	**5**	
**2**	19	1	0	0	20 (51,3%)
**3**	1	6	1	0	8 (20,5%)
**4**	0	1	9	0	10 (25,6%)
**5**	0	0	0	1	1 (2,6%)
**Total (%)**	20 (51,3%)	8 (20,5%)	10 (25,6%)	1 (2,6%)	39

Table S21: Crosstab for inter-reader agreement between radiologist 1 and radiologist 3.

	Radiologist2_withDCE				Total (%)
**Radiologist3_withDCE**	**2**	**3**	**4**	**5**	
**2**	20	0	0	0	20 (51,3%)
**3**	5	3	0	0	8 (20,5%)
**4**	1	1	8	0	10 (25,6%)
**5**	0	0	0	1	1 (2,6%)
**Total (%)**	26 (66,7%)	4 (10,3%)	8 (20,5%)	1 (2,6%)	39

Table S22: Crosstab for inter-reader agreement between radiologist 2 and radiologist 3.

Crosstabs for bpMRI-based PI-RADS v2.1 considering non-PCa lesions (n = 39)

	Radiologist1_noDCE				Total (%)
Radiologist2_noDCE	2	3	4	5	
2	20	7	0	0	27 (69,2%)
3	0	5	1	0	6 (15,4%)
4	0	3	2	0	5 (12,8%)
5	0	0	0	1	1 (2,6%)
Total (%)	20 (51,3%)	15 (38,5%)	3 (7,7%)	1 (2,6%)	39

Table S23: Crosstab for inter-reader agreement between radiologist 1 and radiologist 2.

	Radiologist1_noDCE				Total (%)
Radiologist3_noDCE	2	3	4	5	
2	19	1	0	0	20 (51,3%)
3	1	11	1	0	13 (33,3%)
4	0	3	2	0	5 (12,8%)
5	0	0	0	1	1 (2,6%)
Total (%)	20 (51,3%)	15 (38,5%)	3 (7,7%)	1 (2,6%)	39

Table S24: Crosstab for inter-reader agreement between radiologist 1 and radiologist 3.

	Radiologist2_noDCE				Total (%)
Radiologist3_noDCE	2	3	4	5	
2	20	0	0	0	20 (51,3%)
3	7	6	0	0	13 (33,3%)
4	0	0	5	0	5 (12,8%)
5	0	0	0	1	1 (2,6%)
Total (%)	27 (69,2%)	6 (15,4%)	5 (12,8%)	1 (2,6%)	39

Table S25: Crosstab for inter-reader agreement between radiologist 2 and radiologist 3.

Crosstabs for inter-method agreement

Crosstabs for agreement between mpMRI- and bpMRI-based PI-RADS v2.1 considering all lesions (n = 117)

	Radiologist1_withDCE				Total (%)
Radiologist1_noDCE	2	3	4	5	
2	25	2	0	0	27 (23,1%)
3	0	15	28	0	43 (36,8%)
4	0	0	15	0	15 (12,8%)
5	0	0	0	32	32 (27,4%)
Total (%)	25 (21,4%)	17 (14,5%)	43 (36,8%)	32 (27,4%)	117

Table S26: Crosstab for inter-method agreement for radiologist 1.

	Radiologist2_withDCE				Total (%)
Radiologist2_noDCE	2	3	4	5	
2	35	1	0	0	36 (30,8%)
3	0	8	20	0	28 (23,9%)
4	0	0	21	0	21 (17,9%)
5	0	0	0	32	32 (27,4%)
Total (%)	35 (29,9%)	9 (7,7%)	41 (35,0%)	32 (27,4%)	117

Table S27: Crosstab for inter-method agreement for radiologist 2.

	Radiologist3_withDCE				Total (%)
Radiologist3_noDCE	2	3	4	5	
2	27	1	0	0	28 (23,9%)
3	0	14	21	0	35 (29,9%)
4	0	0	22	0	22 (18,8%)
5	0	0	0	32	32 (27,4%)
Total (%)	27 (23,1%)	15 (12,8%)	43 (36,8%)	32 (27,4%)	117

Table S28: Crosstab for inter-method agreement for radiologist 3.

Crosstabs for agreement between mpMRI- and bpMRI-based PI-RADS v2.1 considering all PCa lesions (n = 78)

	**Radiologist1_withDCE**				**Total (%)**
**Radiologist1_noDCE**	**2**	**3**	**4**	**5**	
**2**	5	2	0	0	7 (9,0%)
**3**	0	7	21	0	28 (35,9%)
**4**	0	0	12	0	12 (15,4%)
**5**	0	0	0	31	31 (39,7%)
**Total (%)**	5 (6,4%)	9 (11,5%)	33 (42,3%)	31 (39,7%)	78

Table S29: Crosstab for inter-method agreement for radiologist 1.

	**Radiologist2_withDCE**				**Total (%)**
**Radiologist2_noDCE**	**2**	**3**	**4**	**5**	
2	9	0	0	0	9 (11,5%)
3	0	5	17	0	22 (28,2%)
4	0	0	16	0	16 (20,5%)
5	0	0	0	31	31 (39,7%)
**Total (%)**	9 (11,5%)	5 (6,4%)	33 (42,3%)	31 (39,7%)	78

Table S30: Crosstab for inter-method agreement for radiologist 2.

	**Radiologist3_withDCE**				**Total (%)**
**Radiologist3_noDCE**	**2**	**3**	**4**	**5**	
**2**	7	1	0	0	8 (10,3%)
**3**	0	6	16	0	22 (28,2%)
**4**	0	0	17	0	17 (21,8%)
**5**	0	0	0	31	31 (39,7%)
**Total (%)**	7 (9,0%)	7 (9,0%)	33 (42,3%)	31 (39,7%)	78

Table S31: Crosstab for inter-method agreement for radiologist 3

Crosstabs for agreement between mpMRI- and bpMRI-based PI-RADS v2.1 considering clinically significant PCa lesions (n = 41)

	**Radiologist1_withDCE**			**Total (%)**
**Radiologist1_noDCE**	**3**	**4**	**5**	
**3**	3	9	0	12 (29,3%)
**4**	0	7	0	7 (17,1%)
**5**	0	0	22	22 (53,7%)
**Total (%)**	3 (7,3%)	16 (39,0%)	22 (53,7%)	41

Table S32: Crosstab for inter-method agreement for radiologist 1.

	**Radiologist2_withDCE**				**Total (%)**
**Radiologist2_noDCE**	**2**	**3**	**4**	**5**	
**2**	1	0	0	0	1 (2,4%)
**3**	0	3	5	0	8 (19,5%)
**4**	0	0	10	0	10 (24,4%)
**5**	0	0	0	22	22 (53,7%)
**Total (%)**	1 (2,4%)	3 (7,3%)	15 (36,6%)	22 (53,7%)	41

Table S33: Crosstab for inter-method agreement for radiologist 2.

	**Radiologist3_withDCE**			**Total (%)**
**Radiologist3_noDCE**	**3**	**4**	**5**	
**3**	4	4	0	8 (19,5%)
**4**	0	11	0	11 (26,8%)
**5**	0	0	22	22 (53,7%)
**Total (%)**	4 (9,8%)	15 (36,6%)	22 (53,7%)	41

Table S34: Crosstab for inter-method agreement for radiologist 3.

Crosstabs for agreement between mpMRI- and bpMRI-based PI-RADS v2.1 considering non-PCa lesions (n = 39)

	Radiologist1_withDCE				Total (%)
**Radiologist1_noDCE**	**2**	**3**	**4**	**5**	
**2**	20	0	0	0	20 (51,3%)
**3**	0	8	7	0	15 (38,5%)
**4**	0	0	3	0	3 (7,7%)
**5**	0	0	0	1	1 (2,6%)
**Total (%)**	20 (51,3%)	8 (20,5%)	10 (25,6%)	1 (2,6%)	39

Table S35: Crosstab for inter-method agreement for radiologist 1.

	Radiologist2_withDCE				Total (%)
**Radiologist2_noDCE**	**2**	**3**	**4**	**5**	
**2**	26	1	0	0	27 (69,2%)
**3**	0	3	3	0	6 (15,4%)
**4**	0	0	5	0	5 (12,8%)
**5**	0	0	0	1	1 (2,6%)
**Total (%)**	26 (66,7%)	4 (10,3%)	8 (20,5%)	1 (2,6%)	39

Table S36: Crosstab for inter-method agreement for radiologist 2.

	Radiologist3_withDCE				Total (%)
**Radiologist3_noDCE**	**2**	**3**	**4**	**5**	
**2**	20	0	0	0	20 (51,3%)
**3**	0	8	5	0	13 (33,3%)
**4**	0	0	5	0	5 (12,8%)
**5**	0	0	0	1	1 (2,6%)
**Total (%)**	20 (51,3%)	8 (20,5%)	10 (25,6%)	1 (2,6%)	39

Table S37: Crosstab for inter-method agreement for radiologist 3.

Crosstabs for agreement between mpMRI- and bpMRI-based PI-RADS v2.1 considering all PCa lesions in PZ (n = 66)

	**Radiologist1_withDCE**				**Total (%)**
**Radiologist1_noDCE**	**2**	**3**	**4**	**5**	
**2**	4	2	0	0	6 (9,1%)
**3**	0	7	18	0	25 (37,9%)
**4**	0	0	10	0	10 (15,2%)
**5**	0	0	0	25	25 (37,9%)
**Total (%)**	4 (6,1%)	9 (13,6%)	28 (42,4%)	25 (37,9%)	66

Table S38: Crosstab for inter-method agreement for radiologist 1.

	**Radiologist2_withDCE**				**Total (%)**
**Radiologist2_noDCE**	**2**	**3**	**4**	**5**	
2	8	0	0	0	8 (12,1%)
3	0	4	15	0	19 (28,8%)
4	0	0	14	0	14 (21,2%)
5	0	0	0	25	25 (37,9%)
**Total (%)**	8 (12,1%)	4 (6,1%)	29 (43,9%)	25 (37,9%)	66

Table S39: Crosstab for inter-method agreement for radiologist 2.

	**Radiologist3_withDCE**				**Total (%)**
**Radiologist3_noDCE**	**2**	**3**	**4**	**5**	
**2**	6	1	0	0	7 (10,6%)
**3**	0	6	16	0	19 (28,8%)
**4**	0	0	17	0	15 (22,7%)
**5**	0	0	0	25	25 (37,9%)
**Total (%)**	6 (9,1%)	6 (9,1%)	29 (43,9%)	25 (37,9%)	66

Table S40: Crosstab for inter-method agreement for radiologist 3
